# Structural basis for TNIP1 binding to FIP200 during mitophagy

**DOI:** 10.1016/j.jbc.2024.107605

**Published:** 2024-07-25

**Authors:** Shengmei Wu, Mingwei Li, Lei Wang, Lingna Yang, Jing Cui, Fudong Li, Qian Wang, Yunyu Shi, Mengqi Lv

**Affiliations:** 1 Hefei National Research Center for Cross Disciplinary Science, School of Life Sciences, Division of Life Sciences and Medicine, University of Science and Technology of China, Hefei, Anhui, China; 2 Ministry of Education Key Laboratory for Membraneless Organelles and Cellular Dynamics, University of Science & Technology of China, Hefei, Anhui, China; 3 Division of Life Sciences and Medicine, Center for Advanced Interdisciplinary Science and Biomedicine of IHM, University of Science and Technology of China, Hefei, Anhui, China; 4 Department of Physics, University of Science and Technology of China, Hefei, Anhui, China; 5 Department of Hepatobiliary Surgery, Innovative Institute of Tumor Immunity and Medicine (ITIM), Anhui Province Key Laboratory of Tumor Immune Microenvironment and Immunotherapy, The First Affiliated Hospital of Anhui Medical University, Hefei, Anhui, China

**Keywords:** crystal structure, focal adhesion kinase (FAK)-interacting protein of 200 kDa (FIP200), TNFAIP3-interacting protein 1 (TNIP1), phosphorylation, mitophagy

## Abstract

TNIP1 has been increasingly recognized as a security check to finely adjust the rate of mitophagy by disrupting the recycling of the Unc-51–like kinase complex during autophagosome formation. Through tank-binding kinase 1–mediated phosphorylation of the TNIP1 FIP200 interacting region (FIR) motif, the binding affinity of TNIP1 for FIP200, a component of the Unc-51–like kinase complex, is enhanced, allowing TNIP1 to outcompete autophagy receptors. Consequently, FIP200 is released from the autophagosome, facilitating further autophagosome expansion. However, the molecular basis by which FIP200 utilizes its claw domain to distinguish the phosphorylation status of residues in the TNIP1 FIR motif for recognition is not well understood. Here, we elucidated multiple crystal structures of the complex formed by the FIP200 claw domain and various phosphorylated TNIP1 FIR peptides. Structural and isothermal titration calorimetry analyses identified the crucial residues in the FIP200 claw domain responsible for the specific recognition of phosphorylated TNIP1 FIR peptides. Additionally, utilizing structural comparison and molecular dynamics simulation data, we demonstrated that the C-terminal tail of TNIP1 peptide affected its binding to the FIP200 claw domain. Moreover, the phosphorylation of TNIP1 Ser123 enabled the peptide to effectively compete with the peptide p-CCPG1 (the FIR motif of the autophagy receptor CCPG1) for binding with the FIP200 claw domain. Overall, our work provides a comprehensive understanding of the specific recognition of phosphorylated TNIP1 by the FIP200 claw domain, marking an initial step toward fully understanding the molecular mechanism underlying the TNIP1-dependent inhibition of mitophagy.

Mitophagy, a best well-characterized type of selective autophagy, serves as a vigilant guardian, ensuring the integrity and functionality of the mitochondrion (1, 2). Dysfunction of mitophagy has been implicated in various disorders, from neurodegenerative diseases to metabolic dysfunction (3, 4, 5, 6, 7). A key initial event in mitophagy is the formation of autophagosome, a unique double-membrane organelle that engulfs the cytosolic cargo destined for degradation. This event is initiated by the Unc-51–like kinase (ULK) complex. During this process, the important step is the interaction with autophagy receptors through FIP200, a component of ULK complex (8, 9, 10, 11, 12, 13).

TNIP1 is increasingly recognized as a negative modulator of NF-κB activation in inflammatory signaling and a potential contributor to multiple autoimmune diseases (14, 15, 16). Recent findings revealed that TNIP1 functions as a checkpoint mechanism that fine-tunes the rate of mitophagy by disrupting the recycling of the ULK complex during autophagosome formation (17). TNIP1 interacts with the autophagy receptor TAX1BP1, occupying the ubiquitin (UB)-binding domain and preventing TAX1BP1 from binding to ubiquitinated substrates, resulting in defective mitophagy (17). This inhibition seems to be regulated by the phosphorylation of TNIP1. The TNIP1–TAX1BP1 complex recruits TBK1, which can phosphorylate TNIP1 and activate the FIR motif of TNIP1 (18). The phosphorylation of the TNIP1 FIR motif enhances its binding affinity for FIP200, enabling TNIP1 to compete with other autophagy receptors, such as CCPG1 or Optineurin (17). Consequently, FIP200 is released from the autophagosome, facilitating further autophagosome expansion (17). However, the mechanism by which FIP200 utilizes its claw domain to recognize the phosphorylation status of residues in the TNIP1 FIR motif remains a compelling topic for structural biology research, raising questions about whether the phosphorylation of the FIR motif regulates the interaction between TNIP1 and the FIP200 claw domain.

Here, we determined several crystal structures of the FIP200 claw domain in complex with phosphorylated TNIP1 FIR peptides and elucidated the specific recognition of different phosphorylated TNIP1 by FIP200 claw domain. In addition, according to further structural analysis and MD simulation, we revealed that the C-terminal elongated tail of TNIP1 peptide is flexible and affects the binding between FIP200 claw domain and TNIP1. Moreover, the phosphorylation of Ser123 in TNIP1 contributed to the ability of the TNIP1 peptide to effectively compete with the FIR motif of autophagy receptor CCPG1 for binding to FIP200 claw domain. In conclusion, our structural and *in vitro* interaction analyses provide mechanistic insights into the specific recognition of phosphorylated TNIP1 peptides by FIP200 claw domain and lay a foundation for further understanding the molecular mechanism of mitophagy.

## Results

**Figure 1:**
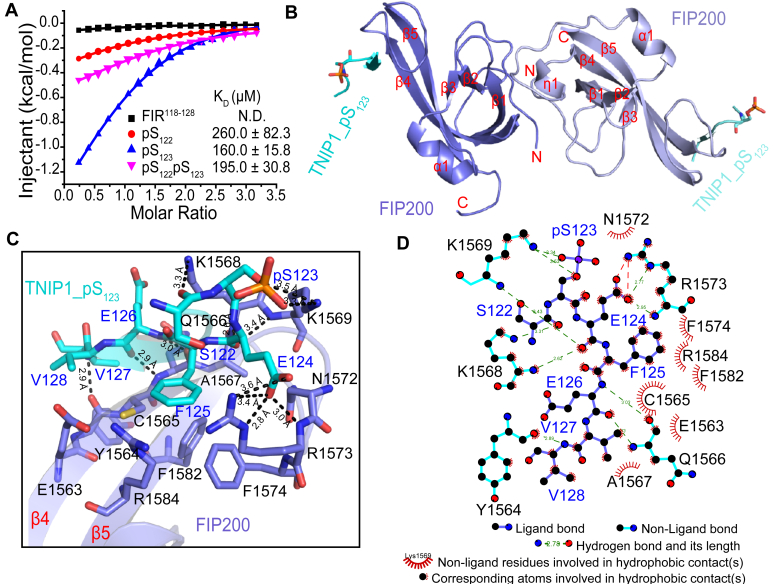
**Structure of FIP200-TNIP1_pS**_**123**_**complex.***A*, the fitting curves of ITC titration data of FIP200 claw domain with different TNIP1 FIR peptides are shown. The unmodified pS_122_, pS_123_, and pS_122_pS_123_ TNIP1 peptides are colored *black*, *red*, *blue*, and *magenta*, respectively. Each *K*_*D*_ value is presented as a fitted value ± SD. *B*, cartoon representation of the overall structure of the dimeric FIP200-TNIP1_pS_123_ complex. The two FIP200 claw domains are colored in *slate* and *light-blue*, while the two TNIP1_pS_123_ peptides are colored in *cyan* and *aquamarine*, respectively. *C*, the detailed interactions between FIP200 claw domain (*slate*, labeled *black*) and the TNIP1_pS_123_ peptide (*cyan*, labeled *blue*). Hydrogen bonds are indicated with *black dotted lines*, with distances measured in Å. *D*, schematic representations of the recognition of TNIP1_pS_123_ (*cyan*, labeled *blue*) by the FIP200 claw domain (*slate*, labeled *black*) produced using the Ligplot program. N. D., no detectable binding.

TBK1 phosphorylates both S122 and S123 in the TNIP1 FIR motif, which is crucial for affecting the binding affinity for FIP200 (18). To investigate this, a series of TNIP1 FIR peptides were synthesized, either unmodified or with phosphorylation on S122 (pS_122_), S123 (pS_123_), or both S122 and S123 (pS_122_pS_123_), respectively. Subsequently, their binding capacity to the FIP200 claw domain was assessed using isothermal titration calorimetry (ITC) assays (Figs. 1*A* and S1, and Table S3). The ITC results revealed that the unmodified TNIP1_FIR peptide showed no detectable binding affinity for the FIP200 claw domain. Phosphorylation on either S122 or S123 enhanced the binding affinity between the FIP200 claw domain and TNIP1 peptides. The binding affinity of FIP200 claw domain to the TNIP1_pS_122_pS_123_ peptide did not increase as expected, when compared with the TNIP1_pS_123_ peptide.

To explore the interactions between TNIP1 and the FIP200 claw domain, we elucidated multiple crystal structures of the complex formed by the FIP200 claw domain and various phosphorylated TNIP1 FIR peptides (Table S1). For the FIP200–TNIP1_pS_123_ complex, in an asymmetric unit, four FIP200 claw molecules assembled into two dimers, with each molecule binding to one TNIP1_pS_123_ peptide (Fig. S2*A*). The two dimers only exhibited an RMSD for Cα atoms of 0.203 Å (185–185 atoms). The homodimerization of the FIP200 claw dimer primarily relies on the interaction between the N-terminal loop of one FIP200 claw domain and the corresponding loop of the other claw monomer. Each FIP200 claw domain within the dimer features a five-stranded antiparallel β-sheet and a short α-helix. A portion of the TNIP1_pS_123_ peptide was observed, forming a short β-strand packing with β4 of the claw domain (Figs. 1*B* and S2, *B* and *C*).

Further structural analysis of the FIP200–TNIP1_pS_123_ complex revealed extensive polar interactions (charge–charge interactions and hydrogen bonding) and hydrophobic interactions (Figs. 1, *C* and *D* and S2*D*). The FIP200 claw domain utilized its positively charged residues, R1573 and K1569 in the loop connecting β4 and β5, to specifically recognize the negatively charged N-terminal of TNIP1_pS_123_ peptide. Specifically, the ε-NH_3_^+^ group of K1569 side chain established specific charge–charge interactions to the phosphate group of TNIP1 pS_123_. R1573 formed specific charge–charge and hydrogen bonding interactions with the side chain of TNIP1 E104. Additionally, the side chains of F125 and V128, located within the conserved LC3 interaction region motif of TNIP1_pS_123_ peptide, inserted into two hydrophobic pockets of FIP200 claw domain. The hydrophobic pocket accommodating F125 included residues C1565, F1574, R1573, F1582, and R1584 of FIP200 claw domain. The side chain of V128 inserted into a small hydrophobic pocket formed by the residues Y1564, Q1566, and C1565 from FIP200 claw domain. Moreover, the ε-NH_3_^+^ group of K1568 side chain formed a hydrogen bond with the carbonyl group of TNIP1 S122 main chain. The main chains of Y1564, C1565, Q1566, and K1568 residues located at β4 of the FIP200 claw domain interacted with the main chains of TNIP1 V109, E107, and E104, forming five backbone hydrogen bonds.

**Figure 2:**
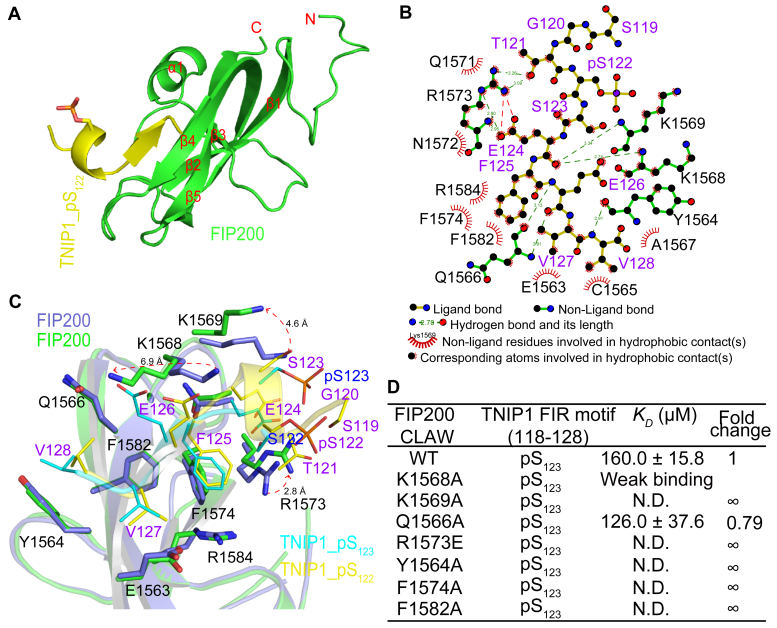
**Structure FIP200-TNIP1_pS**_**122**_**complex.***A*, cartoon representation of one FIP200 claw domain (*green*) in complex with one TNIP1_pS_122_ peptide (*yellow*). *B*, schematic representations of the recognition of TNIP1_pS_122_ (*yellow*, labeled *purple*) by the FIP200 claw domain (*green*, labeled *black*) produced using the Ligplot program. *C*, superposition of the FIP200-TNIP1_pS_123_ complex and FIP200-TNIP1_pS_122_ complex, colored as described in Figure 1, *D* and *E.* The critical residues involved in the recognition are shown in *stick* mode, respectively. Conformational rearrangement of certain residues of the pS_123_-bound FIP200 claw domain relative to those of the pS_122_-bound form. *D*, the binding affinities between TNIP1_pS_123_ peptide with FIP200 claw domain mutants measured by ITC assays.

We also crystallized and determined the structures of the complexes formed by the FIP200 claw domain and the TNIP1_pS_122_ peptide (Table S1). In the FIP200–TNIP1_pS_122_ complex, the FIP200 claw domain adopted a similar conformation to that in the FIP200–TNIP1_pS_123_ complex (Figs. 2*A* and S3). Interestingly, the N-terminal region of the TNIP1_pS_122_ peptide formed a short helix, while the C-terminal region formed a short β-strand that interacted with the β4 of the claw domain (Fig. S3, *C* and *D*). The phosphorylated S122 of TNIP1 peptide was exposed to the solvent and established two hydrogen bonds with TNIP1 S119 instead of forming direct hydrogen bond with K1569 in FIP200 claw domain (Fig. S3*D*), which might lead the binding affinity of FIP200 claw domain to the TNIP1_pS_122_ peptide weaker than the TNIP1_pS_123_ peptide. Molecular interaction patterns between the C-terminal region of TNIP1_pS_122_ and FIP200 claw domain closely resembled those observed with TNIP1_pS_123_ (Figs. 2*B* and S3, *E* and *F*). The superposition of the two complexes revealed significant rearrangement of certain residues in the FIP200 claw domain to facilitate preferential binding to distinct phosphorylated peptides (Fig. 2*C*).

For FIP200–TNIP1_pS_122_pS_123_ complex, due to the electrostatic repulsion between the phosphorylated S122 and S123 residues, the side chain of K1569 did not form additional direct hydrogen bonds with the phosphate group of TNIP1 pS_123_ (Fig. S4). Consequently, the expected enhancement of the binding affinity between the FIP200 claw domain and the TNIP1_pS_122_pS_123_ peptide did not occur as anticipated.

Alanine and glutamine mutations in crucial interface residues of the FIP200 claw domain were introduced, and ITC assays confirmed their significance for TNIP1_pS_123_ peptide binding. As anticipated, the ITC results demonstrated that point mutations in crucial interface residues, including K1569A, R1573E, Y1564A, F1574A, and F1582A of FIP200, significantly abolished the specific interaction between the FIP200 claw domain and TNIP1_pS_123_ peptide (Figs. 2*D* and S5, and Table S3).

**Figure 3:**
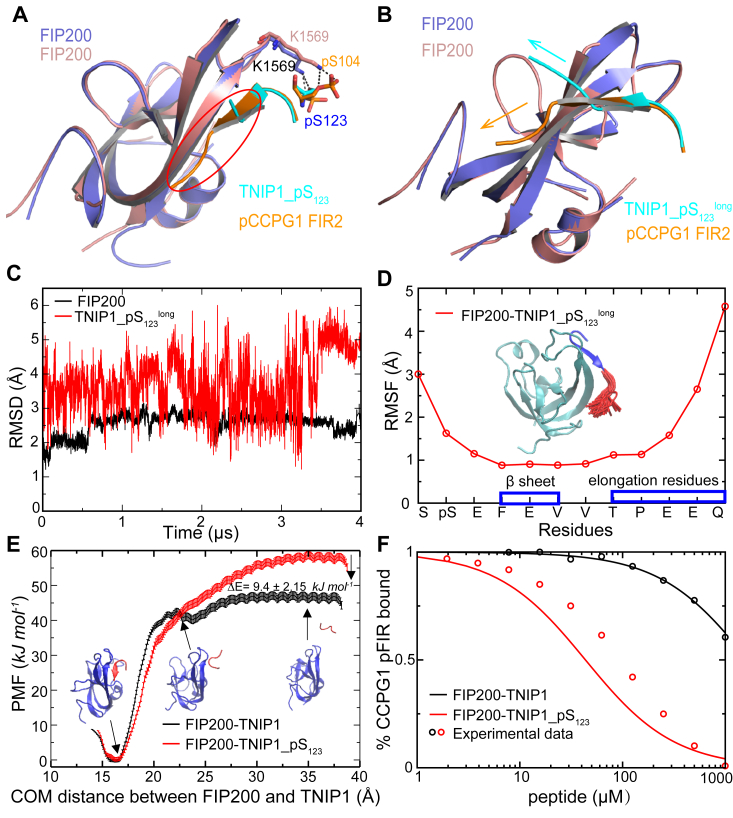
**Biochemical and structural analyses of the FIP200-TNIP1_pS**_**123**_**complex compared with the FIP200/p-CCPG1 FIR2 complex.***A*, superposition of the FIP200-TNIP1_pS_123_ complex and FIP200/p-CCPG1 FIR2 complex showed that the orientations of the C termini of two peptides were different (highlighted in *red*). The FIP200-TNIP1_pS_123_ complex is colored as described in Figure 1*B*. The FIP200 claw domain and p-CCPG1 peptide in FIP200/p-CCPG1 complex are colored *salmon* and *orange*, respectively. *B*, superposition of the FIP200-TNIP1_pS_123_^long^ complex and FIP200/p-CCPG1 FIR2 complex complex, colored as described in (A). The different directions of the C terminus of two peptides were showed with arrows. *C*, conformational changes of FIP200-TNIP1_pS123^long^ complex in molecular dynamics simulations. RMSD evolutions of FIP200 claw domain (*black*) and TNIP1_pS_123_^long^ peptide (*red*). *D*, RMSF of each Cα atom in the TNIP1_pS_123_^long^ peptide. The structure depicts the superposition of 50 elongated tails of TNIP1 in simulations for illustrative purposes. FIP200 claw domain is colored in *cyan*. For TNIP1_pS_123_^long^ peptide, the elongated tail is colored in *red* and the rest are colored in *blue*. *E*, potential of mean force (PMF) profiles as a function of the center-of-mass distance between FIP200 claw domain and unmodified TNIP1 (*black*) and TNIP1_pS_123_ (*red*). *F*, competition of the TNIP1 peptides in displacing p-CCPG1 FIR2 peptides from binding with FIP200 claw domain. The y-axis is presented in logarithmic scale.

Consistent with previous studies, the TNIP1_pS_123_ peptide binds to FIP200 claw domain much weaker than the autophagy receptor p-CCPG1 FIR2 peptide (Table S3). Comparisons between the FIP200–TNIP1_pS_123_ complex and FIP200/p-CCPG1 FIR2 complex (PDB ID: 7D0E) highlighted similarities and differences in binding modes (Fig. 3*A*). The TNIP1 FIR_pS_123_ and p-CCPG1 FIR2 exhibited similar binding modes, but differences in C termini orientations could contribute to varying binding affinities to the FIP200 claw domain. To confirm this, we determined the complex structure of FIP200 claw domain with a novel peptide with an elongated C terminus (TNIP1_pS_123_^long^) (Fig. S6 and Table S1). The complex revealed that the elongated C-terminal residues of the new peptide were exposed to the solvent instead of interacting with β4 of the FIP200 claw domain (Fig. S6*D*). Then, 4-μs MD simulations for the FIP200–TNIP1_pS_123_^long^ complex were conducted. FIP200 claw domain maintains stable throughout the entire simulation, with an RMSD of 2.6 Å (Fig. 3*C*). In contrast, the RMSD of TNIP1_pS_123_^long^ peptide exhibits significant fluctuations between 1.2 and 6.0 Å, indicating its flexibility (Fig. 3*C*). To explore the origin of this flexibility, we calculated the RMSF of each C_α_ atom in the peptide. The analysis revealed that the central beta sheet of the peptide (F125-V127) is relatively stable, while the elongated tail of TNIP1_pS_123_^long^ (T129-Q133) is quite flexible and disordered (Fig. 3*D*), consistent with experimental findings.

Next, we investigated the binding of TNIP1 peptides to FIP200 in distinct states while competing with p-CCPG1 FIR2 peptide. Assuming that in solution, there is a thermodynamic equilibrium as expressed in Equation 1:

(1) $$\begin{array}{c} FIP200-TNIP1\_pS_{123}\overset{u_{2}}{\underset{w_{2}}{⇆}}FIP200\overset{u_{1}}{\underset{w_{1}}{⇄}}FIP200/p-CCPG1\;FIR2 \end{array}$$

where $u_{1}$ and $u_{2}$ represent the rates of forming the FIP200/p-CCPG1 FIR2 and FIP200-TNIP1_pS_123_ binding complexes, respectively, and $w_{1}$ or $w_{2}$ is the corresponding dissociation rate, then the probability of forming FIP200/p-CCPG1 FIR2 ($P_{1}$) can be expressed in the following form:

(2) $$\begin{array}{c} P_{1}=\frac{u_{1}w_{2}}{w_{1}w_{2}+u_{1}w_{2}+u_{2}w_{2}} \end{array}$$

We further assumed that the binding process is diffusion-controlled, meaning that $u_{1}$ and $u_{2}$ are proportional to the concentrations of p-CCPG1 FIR2 and TNIP1_pS_123_, respectively. In addition, $w_{1}$ and $w_{2}$ are determined by the activation barrier in the dissociation free energy profile using the transition state theory. Consequently, the following two relations hold. In the presence of the unmodified TNIP1 peptide, the portion of the FIP200/p-CCPG1 FIR2 binding complex in the solution is:

(3) $$y_{1}=\frac{1}{1+a_{0}\left[\text{TNIP}1\right]}$$

while in the presence of phosphorylated TNIP1_pS_123_, the same portion changes to:

(4) $$y_{2}=\frac{1}{1+a_{0}e^{-\Delta E/k_{B}T}\left[\text{TNIP}1\right]}$$

In those two equations, $a_{0}$ is a constant and ΔE represents the difference in the activation free energy barrier between the unmodified TNIP1 and TNIP1_pS_123_. We initially obtained $a_{0}$ by fitting Equation 3 to the experimental data of the unmodified TNIP1 ($a_{0}$ = 0.0006 μM^−1^). The center of mass (COM) distance between FIP200 and TNIP1 was selected as the reaction coordinate to calculate the binding free energy profile. In this profile, the crystal structure of the FIP200-TNIP1 binding complex is situated at the global free energy minimum, while the unbinding state occurs at a plateau. As the specific COM distance increases, the contact number between FIP200 and TNIP1 gradually diminishes to zero (Fig. S7). These findings collectively suggest that the COM distance serves as a useful reaction coordinate for elucidating the dissociation processes. Additionally, this method has proven highly effective in various applications (19, 20, 21). Subsequently, we computed ΔE by molecular dynamic simulations (Fig. 3*E*, $\Delta E=9.4\;\pm \;2.15\;kJ\;{mol}^{-1}$). Finally, we predicted the portion of the FIP200/p-CCPG1 FIR2 binding complex in the presence of TNIP1_pS_123_, $y_{2}$, by substituting $a_{0}$ and ΔE into Equation 4. The theoretical prediction matches with the experimental measurement qualitatively well (Fig. 3*F*), validating the theoretical model. These data collectively support that TNIP1_pS_123_ can effectively compete with p-CCPG1 FIR2 for binding, attributed to the enhanced binding affinity following phosphorylation compared to the WT (Fig. 3*E*).

## Discussion

As a negative regulator of mitophagy, TNIP1 competes with autophagy receptors for binding to FIP200, releasing FIP200 from the ULK complex and thereby disrupting autophagosome formation (17). Here, we elucidated the molecular mechanism underlying the interaction between FIP200 claw domain and phosphorylated TNIP1 peptides. Moreover, we demonstrated that phosphorylated TNIP1 FIR can bind competitively to the FIP200 claw domain with the FIR motifs of other autophagy receptors. Previous studies have proposed two different binding patterns of FIP200 claw domain interact with the FIR motifs of autophagy receptors (22, 23). One suggests that the FIP200 claw domain can directly bind to FIR motifs, with phosphorylation in FIR motif significantly enhancing its affinity for FIP200 claw domain, such as FIP200 and CCPG1 FIR2. The other suggests that the recognition between FIP200 claw domain and some autophagy receptors depends on the phosphorylation of FIR motifs, with an increase in the amount of phosphorylation in TNIP1 FIR enhancing its affinity for FIP200 claw domain, such as FIP200 and Optineurin. Our findings revealed that the recognition between FIP200 claw domain and TNIP1 FIR is not consistent with these two patterns. The recognition of the FIP200 claw domain with TNIP1 occurs only when the FIR motif of TNIP1 is phosphorylated, even though the residue preceding the LC3-interacting region motif of TNIP1 is already an acidic Glu. We suspected that this was due to the effect of the C-terminal tail of TNIP1 FIP. Nevertheless, the recognition between FIP200 claw domain and TNIP1 FIR is in line with the functional requirement of TNIP1 as a mitophagy inhibitory factor. Only when TBK1 phosphorylates the TNIP1 FIR can TNIP1 effectively compete with autophagy receptors for binding to FIP200 and inhibit the early stages of autophagosome biogenesis. In addition, unlike pattern II, the cumulative phosphorylation of the TNIP1 FIR motif does not enhance its binding affinity to FIP200 claw domain. This may because excessive phosphorylation in TNIP FIR leads to electrostatic repulsion and structural instability, as shown in FIP200–TNIP1_pS_122_pS_123_ complex.

In addition to competing with other autophagy factors for binding to FIP200, TNIP1 is involved in the inhibition of mitophagy through a series of protein interactions, such as interactions with LC3/GABARAP and TAX1BP1. From a structural biology perspective, systematically elucidating the interaction network of TNIP1 can facilitate a deeper understanding of the molecular mechanisms by which TNIP1 acts as a safety checkpoint to finely tune the rate of mitophagy.

## Experimental procedures

### Protein expression and purification

DNA fragment of human FIP200 claw domain_1490–1594_ was cloned into a modified pET28a (Novagen) plasmid, which contained an N-terminal SUMO-tag and a ULP1 protease cleavage site. All mutants were generated using a MutanBEST Kit (TaKaRa) and confirmed by DNA sequencing. The proteins were expressed in *Escherichia coli* Rosetta (DE3) cells (Novagen) cultured in LB medium at 37 °C to A_600_ = 1.0 and then shifted to 16 °C for 24 h after induction with 0.2 mM IPTG. Bacterial pellets were resuspended in buffer A (50 mM NaH_2_PO_4_ and 1 M NaCl, pH 7.0) and lysed by sonication. The crude lysate was then centrifuged at 14,000 rpm for 30 min at 4 °C. The supernatant was applied to a Ni-NTA column (QIAGEN), followed by size-exclusion chromatography using a Superdex 200 (GE Healthcare) column. After cleavage with ULP1 protease overnight at 16 °C to remove the SUMO-tag, an additional purification step was performed with a HiTrap SP FF column. The purified protein was concentrated to ∼20 mg/ml in buffer B (50 mM NaH_2_PO_4_, 150 mM NaCl, pH 7.0) and stored at −80 °C.

### Isothermal titration calorimetry

ITC assays were performed on a Microcal PEAQ-ITC instrument (Malvern) at 20 °C. The concentrations of proteins were determined spectrophotometrically. Proteins and peptides were dialyzed against buffer B and adjusted to 0.25 mM and 4 mM, respectively. The titration protocol was the same for all the measurements, composed of a single initial injection of 1 μl peptide, followed by 19 injections of 2 μl peptide into protein samples, the intervals between injections were set to 120 s, and the reference power was 5 μcal ·s^−1^. Thermodynamic data were analyzed with a single binding site model using MicroCal PEAQ-ITC Analysis Software provided by the manufacturer (Malvern). The thermodynamic parameters of the ITC experiments are listed in Table S3.

### Crystallization, data collection, and structure determination

The FIP200 proteins and different TNIP1 FIR peptides were mixed at a 1:5 M ratio at a final concentration of 15 mg/ml, respectively. Crystals of the FIP200 claw-TNIP1_pS_123_ complex were grown at 16 °C using the sitting drop vapor diffusion method by mixing 1 μl of mixture with 1 μl of reservoir buffer (0.018 M magnesium chloride hexahydrate, 0.1 M imidazole, 0.1 M MES, 0.1 M monohydrate, 10% MPD, 10% PEG1000, 10% PEG3350, pH 6.5). The FIP200 claw-TNIP1_pS_122_ complex, FIP200 claw-TNIP1 _ pS_122_pS_123_ complex, and FIP200-TNIP1_pS_123_^long^ complex were crystallized in 25% v/v PEG smear medium, 0.1 M sodium cacodylate, 0.2 M ammonium sulfate, pH 5.5; 15% w/v PEG 6000, 0.1 M MES, 5% v/v MPD, pH 6.5; and 0.2 M imidazole malate, 42% PEG 600, respectively, at 20 °C by vapor diffusion in hanging drops.

Crystals were soaked in mother liquor supplemented with 20% glycerol before being flash-frozen in liquid nitrogen. Datasets were collected on beamline 19U1 of the Shanghai Synchrotron Radiation Facility. The data were processed with XDS and programs in the CCP4 suite. The complex structure of FIP200 claw-TNIP1_pS_123_ was solved by molecular replacement with the program MOLREP (24), using the FIP200 claw structure (PDB ID: 6DCE) as the search model. The FIP200 claw-TNIP1_pS_123_ structure was subsequently used as the search model for molecular replacement using program MOLREP for other complexes. The TNIP1 FIR peptides were modeled in COOT (25). All initial models were refined using the maximum likelihood method implemented in REFMAC5 (26) as part of the CCP4i program suite and rebuilt interactively using the program COOT. All the structures were finally refined by PHENIX (27). Crystallographic parameters are listed in Table S1. The B-values of each amino acid in the TNIP1_pS_122_, TNIP1_pS_123_, and TNIP1_pS_122_pS_123_ peptides are listed in Table S2. All images of the structures were prepared using PyMOL (http://www.pymol.org/).

### All-atom MD simulations

For FIP200–TNIP1_pS_123_^long^ complex, we added the last three residues E131, E132, and Q133 of the C-terminal and the missing residues 1543 to 1550 of FIP200 by Modeller (28). The proteins were described with the AMBER ff14SB force filed (29). The systems were solvated in a cubic box with the periodic boundary condition. The minimum distance between the solute and the box boundary was 20 Å. The box was filled with TIP3P water molecules (30). Na^+^ and Cl^−^ were added into the box, in order to neutralize the overall charge of the protein and achieve a salt concentration of 150 mM, aligning with the experimental conditions. The cutoff for van der Waals interactions was 10 Å. The long-range electrostatic interactions were calculated using the PME method (31), with a 10 Å cutoff for the direct space sums, a 0.12 nm FFT grid spacing, and a 4-order interpolation polynomial for the reciprocal space sums. Temperature was maintained at 300 K using the V-rescale Thermostat (32). Pressure was maintained at 1 bar by Berendsen algorithm (33). Nonhydrogen bonds were constrained using the SHAKE algorithm (34). The 4-μs MD simulations were performed for FIP200-TNIP1_pS_123_^long^ system by utilizing Gromacs-2023 (35), with a 2-fs integration step.

### Free energy calculations

Umbrella sampling (36, 37, 38) was used to calculate the dissociation free energy of TNIP1 peptide from FIP200. First, we performed well-tempered metadynamics (39) to trace the dissociation pathway as a function of the center-of-mass distance between FIP200 and TNIP1 (*D*_*com*_). The bias factor was set to 20, and the value of sigma was set to 0.05. Next, the structures along the pathway were saved and served as the initial structure for the subsequent umbrella sampling. There are a total of 33 windows, with a spacing of 0.5 Å from *D*_*com*_ = 15 Å to *D*_*com*_ = 25 Å and 1.0 Å from *D*_*com*_ = 25 Å to *D*_*com*_ = 38 Å. We performed 100-ns simulations at each window, which resulted in a total simulation time of 3300 ns. The spring constant was set to 10,000 kJ mol^−1^ nm^−2^. For free energy calculation between unmodified TNIP1 peptide and FIP200, we removed the phosphorylation group of S123 in TNIP1 peptide in MD simulation.

The trajectories were analyzed using the weighted histogram analysis method (40) to derive potential of mean force as a function of the COM distance, and the error was estimated using the bootstrap method (41).

## Data availability

The coordinates and structure factors for the structures of FIP200 claw were deposited into the Protein Data Bank under accession codes 8YFK, 8YFL, 8YFM, and 8YFN.

## Supporting information

This article contains supporting information.

## Conflict of interest

The authors declare that they have no conflicts of interest with the content of this article.
