# Supplementary material for: Structural basis for TNIP1 binding to FIP200 during mitophagy
Source: J Biol Chem. 2024 Jul 25;300(8):107605. doi: 10.1016/j.jbc.2024.107605 (PMC11367644; doi:10.1016/j.jbc.2024.107605)
Supplement: Supplemental Figures S1–S7 and Tables S1–S4 [file mmc1.docx]

**Supplementary Materials**

**Structural basis for TNIP1 binding to FIP200 during mitophagy**

Shengmei Wu^1,2,3†^, Mingwei Li^4†^, Lei Wang^1,2,3^, Lingna Yang^1,2,3^, Jing Cui^1,2,3^, Fudong Li^1,2,3^, Qian Wang^4*^, Yunyu Shi^1,2,3*^, Mengqi Lv^1,2,3,5†*^

1. Hefei National Research Center for Cross Disciplinary Science, School of Life Sciences, Division of Life Sciences and Medicine, University of Science and Technology of China, Hefei, Anhui 230026, China.

2. Ministry of Education Key Laboratory for Membraneless Organelles and Cellular Dynamics, University of Science & Technology of China, Hefei, Anhui 230026, China.

3. Center for Advanced Interdisciplinary Science and Biomedicine of IHM, Division of Life Sciences and Medicine, University of Science and Technology of China, Hefei, Anhui 230026, China

4. Department of Physics, University of Science and Technology of China, Hefei, Anhui 230026, China

5. Department of Hepatobiliary Surgery, Innovative Institute of Tumor Immunity and Medicine (ITIM), Anhui Province Key Laboratory of Tumor Immune Microenvironment and Immunotherapy, The First Affiliated Hospital of Anhui Medical University, Hefei, 230022, China

^*^ To whom correspondence should be addressed. Mengqi Lv, Tel: +86 551 63606364; Fax: +86 551 63601 443; Email: lmq123@ustc.edu.cn

Correspondence may also be addressed to Yunyu Shi, Tel: +86 551 63607464; Fax: +86 551 6360 1443; Email: yyshi@ustc.edu.cn

Correspondence may also be addressed to Qian Wang; Email: wqq@ustc.edu.cn

^†^ The authors wish it to be known that, in their opinion, these authors contributed equally.

**Supplementary Figure Legends**

**Supplementary Figure S1.** The raw ITC titration data of FIP200 claw domain with different TNIP1 FIR peptides and their fitting curves are shown. Each *K_D_* value is presented as a fitted value ± SD. N. D., no detectable binding.

**Supplementary Figure S2.** Structural overview of FIP200 claw domain in complex with TNIP1_pS_123_ peptide. (A) Symmetry and stereo views of the FIP200-TNIP1_pS_123_ complex in an asymmetric unit. (B) The electrostatic surface representation of FIP200 claw domain in complex with TNIP1_pS_123_ (sticks mode in cyan). The positively charged, negatively charged and neutral areas are represented in blue, red and white, respectively. The 2*Fo*-*Fc* electron-density map of the TNIP1_pS_123_ peptide was contoured at 1.0 σ (yellow). (C) The *Fo–Fc* electron density map of TNIP1_pS_123_ peptide is shown (contoured at 2.0 σ). (D) Higher magnification views of individual interactions between FIP200 claw domain (slate, labeled black) and TNIP1_pS_123_ peptide (cyan, labeled blue). Hydrogen bonds are indicated with black dotted lines, with distances measured in Å.

**Supplementary Figure S3.** Structural overview of FIP200 claw domain in complex with TNIP1_pS_122_ peptide. (A) Symmetry and stereo views of the FIP200-TNIP1_pS_122_ complex in an asymmetric unit. (B) The electrostatic surface representation of FIP200 claw domain in complex with TNIP1_pS_122_ (sticks mode in yellow). The positively charged, negatively charged and neutral areas are represented in blue, red and white, respectively. The 2*Fo*-*Fc* electron-density map of the TNIP1_pS_122_ peptide was contoured at 1.0 σ (yellow). (C) The *Fo–Fc* electron density map of TNIP1_pS_122_ peptide is shown (contoured at 2.0 σ). (D) The detailed interactions within the C-terminal helix of TNIP1_pS_122_ peptide. (E) The detailed interactions between FIP200 claw domain (green, labeled black) and the TNIP1_pS_122_ peptide (yellow, labeled purple). Hydrogen bonds are indicated with black dotted lines, with distances measured in Å. (F) Superposition of the FIP200-TNIP1_pS_123_ complex and FIP200-TNIP1_pS_122_ complex, colored as described in Figure 1D and 1E. FIP200 claw domain in the FIP200-TNIP1_pS_122_ complex adopted a similar conformation with that in the FIP200-TNIP1_pS_123_ complex, displaying an overall r.m.s.d. for Cα atoms of 0.483 Å.

**Supplementary Figure S4.** Structural overview of FIP200 claw domain in complex with TNIP1_pS_122_pS_123_ peptide. (A) Symmetry and stereo views of the FIP200- TNIP1_pS_122_pS_123_ complex in an asymmetric unit. (B) The electrostatic surface representation of FIP200 claw domain in complex with TNIP1_pS_122_pS_123_ (sticks mode in grey). The positively charged, negatively charged and neutral areas are represented in blue, red and white, respectively. The 2*Fo*-*Fc* electron-density map of the TNIP1_pS_122_pS_123_ peptide was contoured at 1.0 σ (yellow). (C) The *Fo–Fc* electron density map of TNIP1_ pS_122_pS_123_ peptide is shown (contoured at 2.0 σ). (D) The detailed interactions between FIP200 claw domain (magenta, labeled black) and the TNIP1_pS_122_pS_123_ peptide (gray, labeled orange). Hydrogen bonds are indicated with black dotted lines, with distances measured in Å. (E) Schematic representations of the recognition of TNIP1_pS_122_pS_123_ (gray, labeled orange) by FIP200 claw domain (magenta, labeled black) produced using the Ligplot program.

**Supplementary Figure S5.** The raw ITC titration data of mutated FIP200 claw domain with TNIP1_pS_123_ peptide and their fitting curves are shown. Each *K_D_* value is presented as a fitted value ± SD. N. D., no detectable binding.

**Supplementary Figure S6.** Structural overview of FIP200 claw domain in complex with TNIP1_pS_123_^long^ peptide. (A) Symmetry and stereo views of the FIP200- TNIP1_pS_123_^long^ complex in an asymmetric unit. (B) The electrostatic surface representation of FIP200 claw domain in complex with TNIP1_pS_123_^long^ (sticks mode in cyan). The positively charged, negatively charged and neutral areas are represented in blue, red and white, respectively. The 2*Fo*-*Fc* electron-density map of the TNIP1_pS_123_^long^ peptide was contoured at 1.0 σ (yellow). (C) The *Fo–Fc* electron density map of TNIP1_pS_123_^long^ peptide is shown (contoured at 2.0 σ). (D) Cartoon representation of one FIP200 claw domain (slate) in complex with one TNIP1_pS_123_^long^ peptide (cyan), in which the elongated C terminus of the peptide is flexible facing to the solvent (highlighted with solid red circle).

**Supplementary Figure S7.** The contact number along the center-of-mass distance between the FIP200 claw domain and TNIP1.

**Supplementary Figures**

**Supplementary** **Figure S1**

**
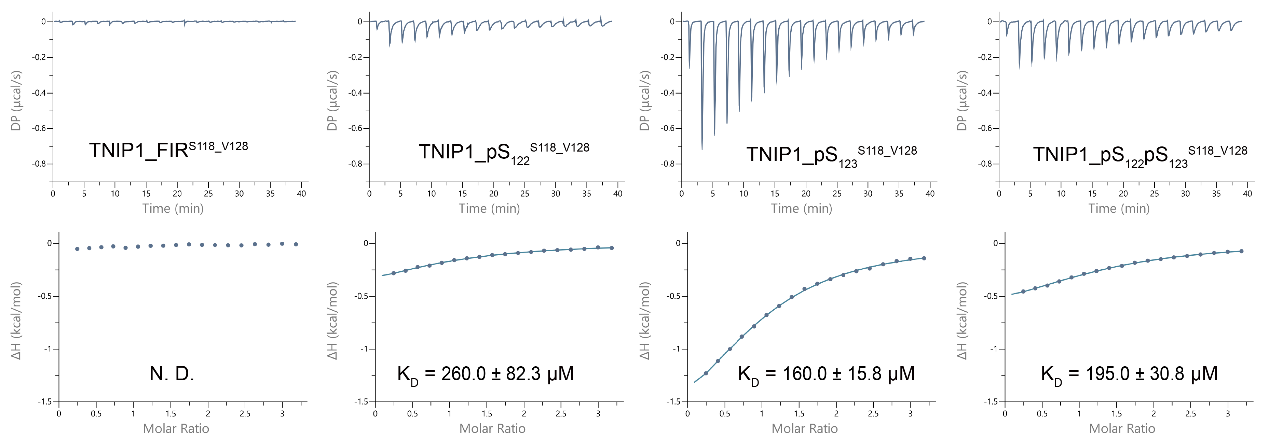
**

**Supplementary Figure S2**

**
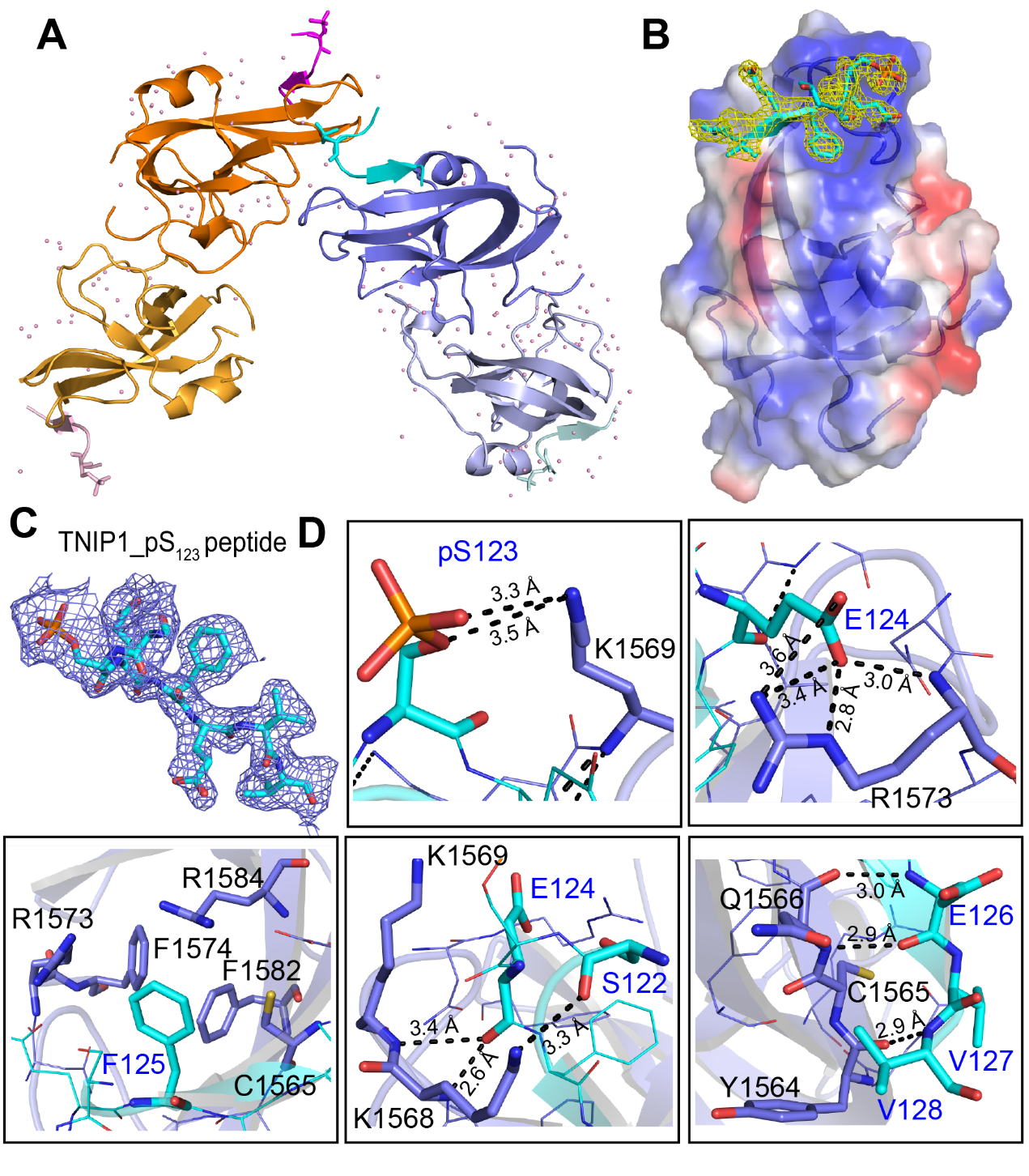
**

**Supplementary Figure S3.**

**
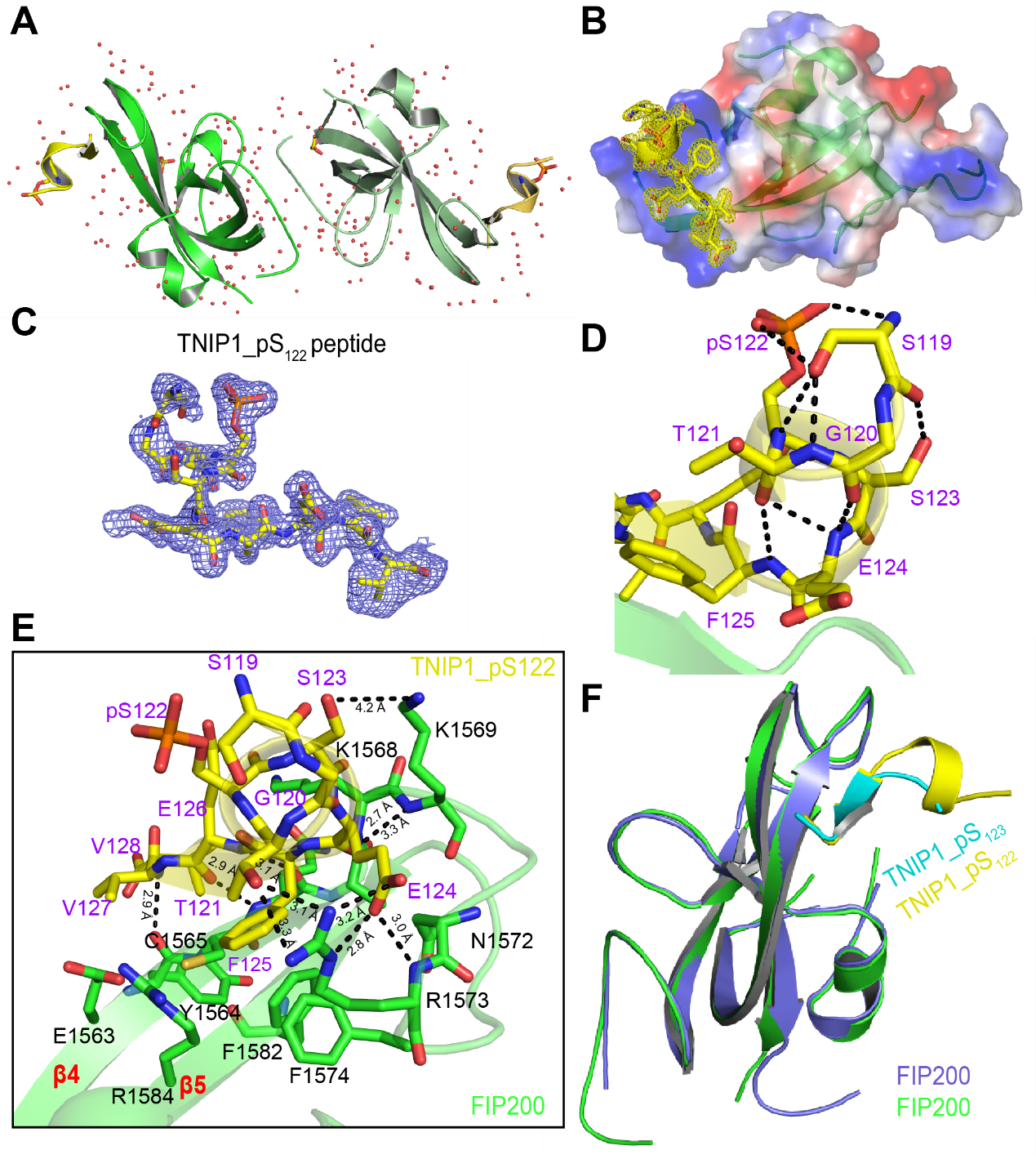
**

**Supplementary Figure S4.**

**
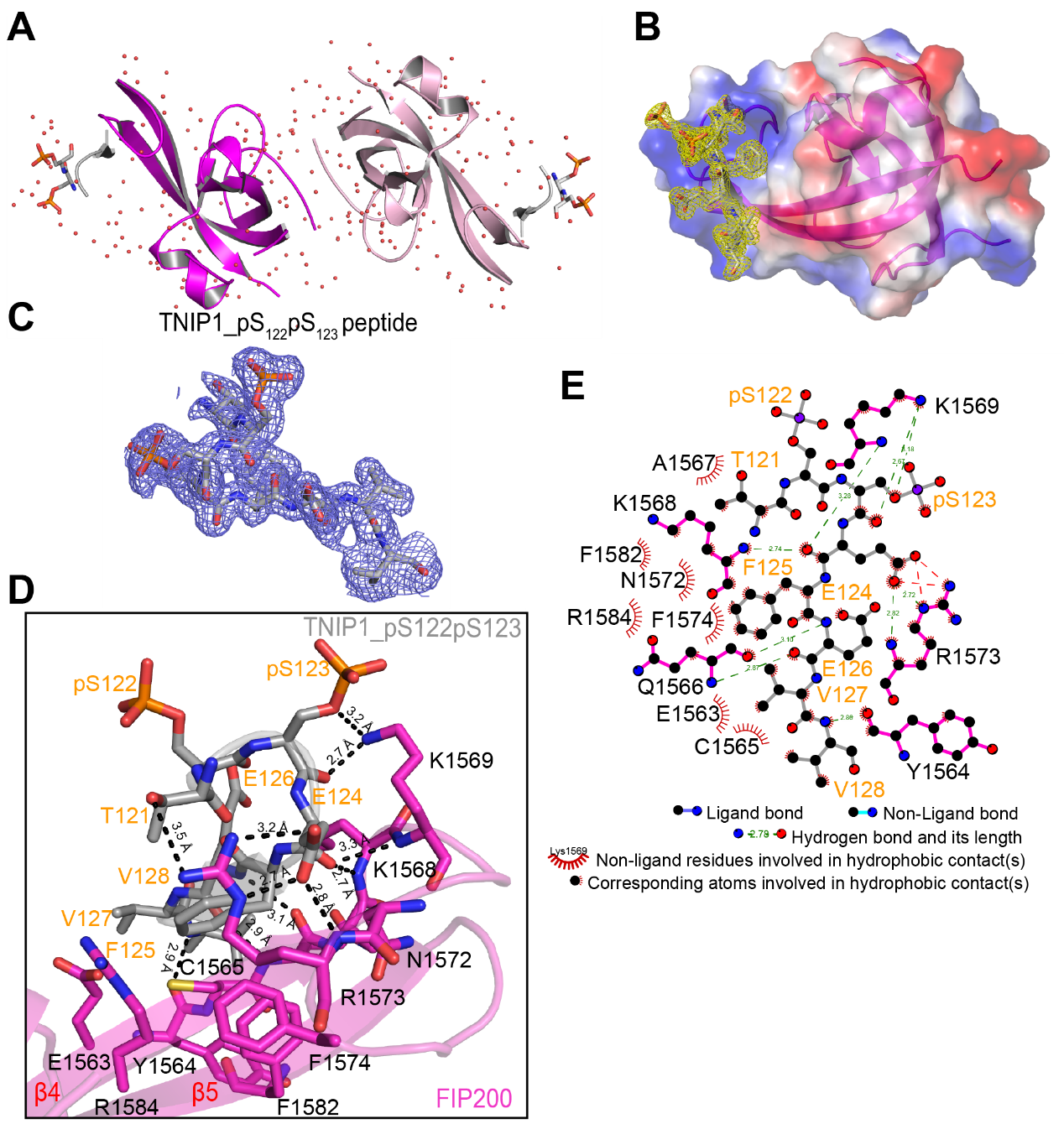
**

**Supplementary Figure S5.**

**
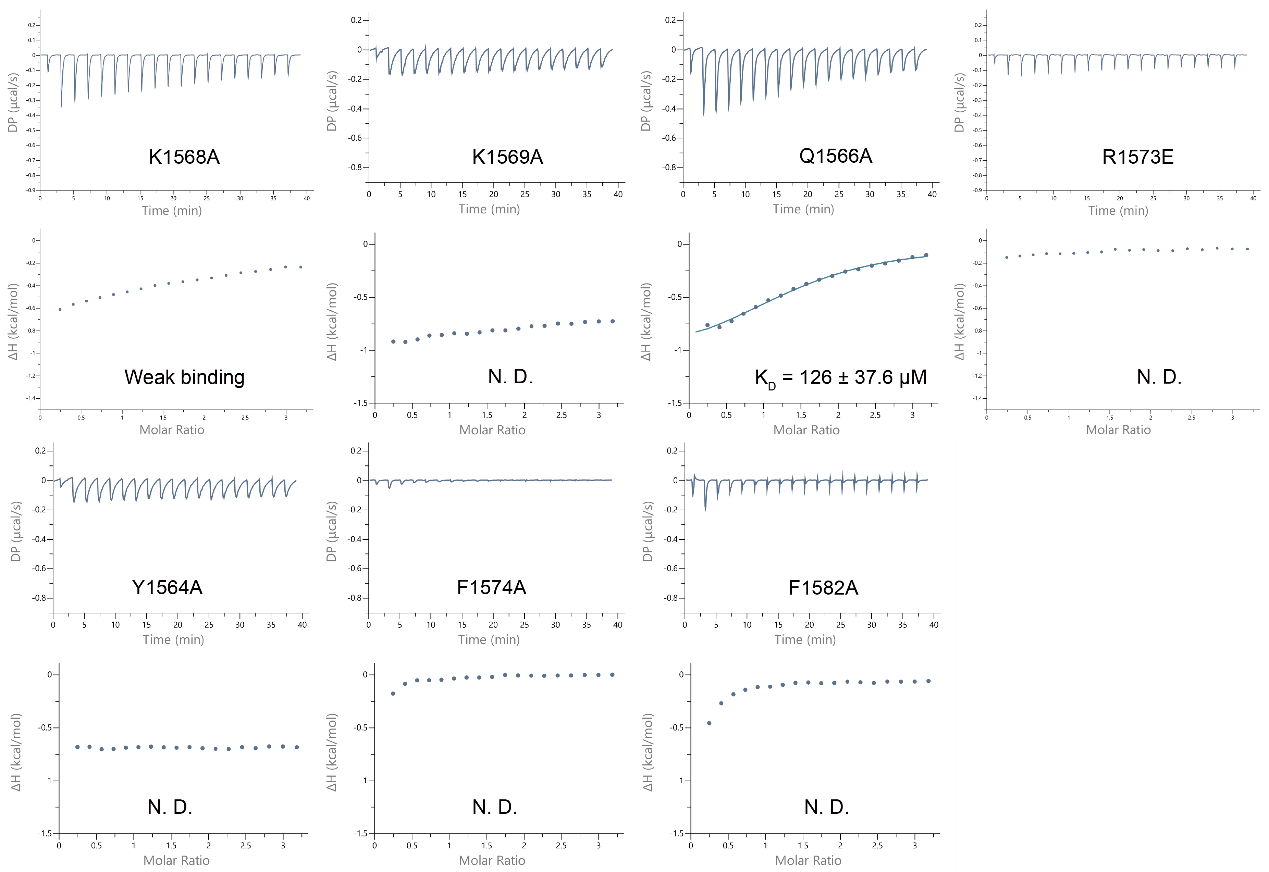
**

**Supplementary Figure S6.**

**
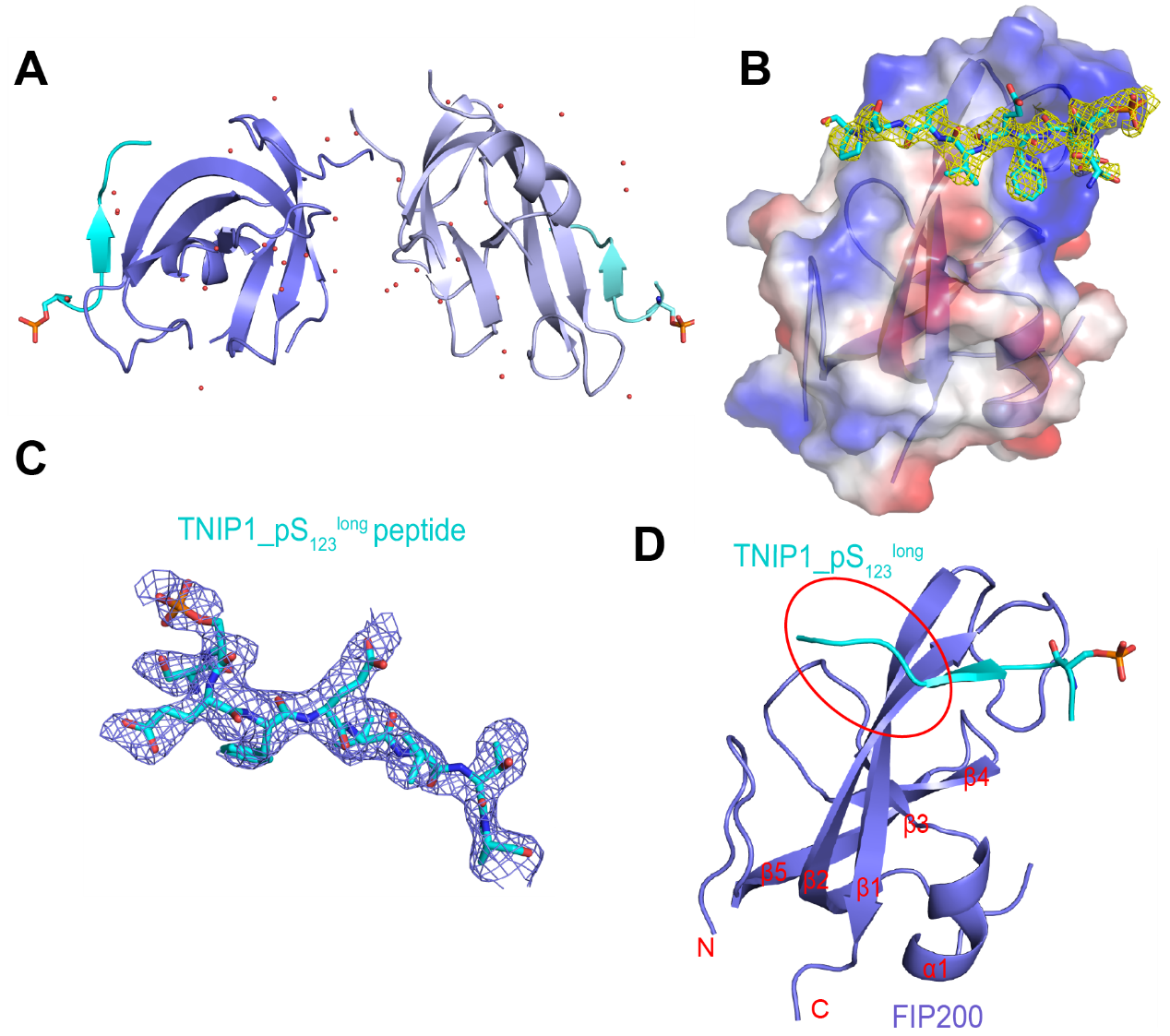
**

**Supplementary Figure S7.**

**
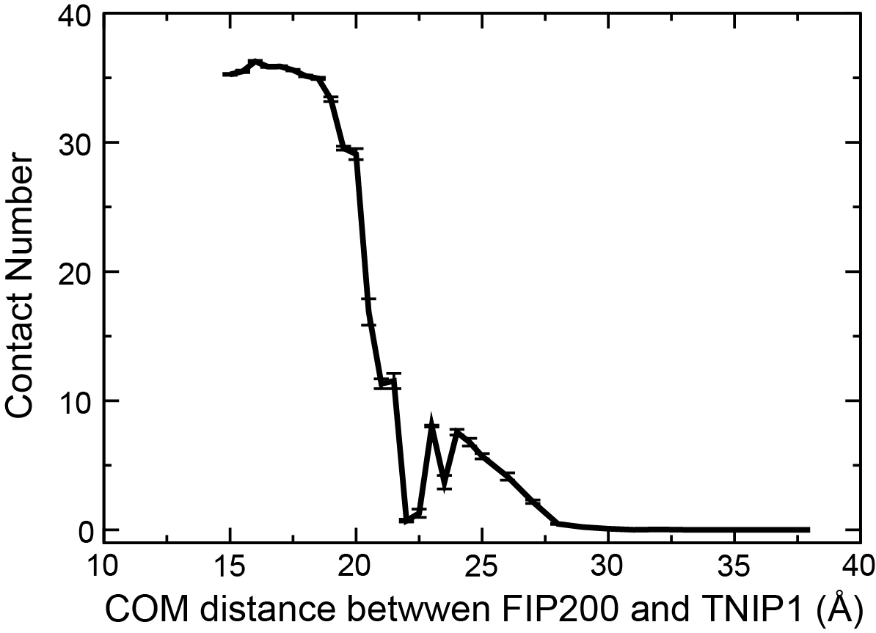
**

**Supplementary** **Tables**

Table S1. Data collection and refinement statistics.

| **Data collection** | FIP200 claw- TNIP1_pS_123_ | FIP200 claw- TNIP1_pS_122_ | FIP200 claw- TNIP1_ pS_122_pS_123_ | FIP200 claw- TNIP1_  pS_123_^long^ |
| --- | --- | --- | --- | --- |
| Beamline  Space group | 19U1, SSRF  *P*1 | 19U1, SSRF  *P*3_2_ | 19U1, SSRF  *P*3_2_ | 19U1, SSRF  *P*4_3_ |
| PDB code | 8YFK | 8YFM | 8YFL | 8YFN |
| Wavelength (Å) | 0.9789 | 0.9789 | 0.9789 | 0.9789 |
| Resolution (Å) | 37.80-2.00 (2.05-2.00)^a^ | 30.54-1.50 (1.53-1.50)^a^ | 32.70-1.50 (1.53-1.50)^a^ | 25.68-2.30 (2.38-2.30)^a^ |
| Cell dimensions |  |  |  |  |
| a, b, c (Å) | 37.81, 45.94, 72.95 | 61.07 61.07 65.30 | 60.92 60.92 65.39 | 51.37 51.37 85.21 |
| α, β, γ (°) | 89.67 88.74 81.20 | 90 90 120 | 90 90 120 | 90 90 90 |
| Unique reflections | 31812 (1488) | 43527 (2108) | 43381 (2075) | 9845 (480) |
| Completeness (%) | 97.2 (95.8) | 99.7 (97.3) | 99.8 (97.7) | 99.7 (99.7) |
| Redundancy | 3.4 (2.1) | 9.6 (6.4) | 9.7 (6.5) | 10.4 (10.3) |
| I/σI | 14.0 (2.9) | 24.8 (2.3) | 19.6 (3.7) | 11.6 (2.6) |
| *R*_merge_ (%) | 4.2 (29.8) | 4.1 (54.8) | 6.3 (31.8) | 12.2 (85.8) |
| CC^1/2^ | 0.994 (0.908) | 0.999 (0.918) | 0.998 (0.966) | 0.998 (0.892) |
| **Refinement** |  |  |  |  |
| *R*_work_ (%) | 18.28 | 19.25 | 20.03 | 18.83 |
| *R*_free_ (%) | 21.96 | 20.88 | 21.77 | 23.64 |
| No. of atoms  Protein | 3313 | 1719 | 1648 | 1618 |
| Water | 177 | 238 | 207 | 38 |
| Average B factors (Å^2^) |  |  |  |  |
| Protein | 42.94 | 34.81 | 40.71 | 61.24 |
| Water | 43.45 | 44.02 | 41.77 | 51.19 |
| Root mean square deviations |  |  |  |  |
| Bond lengths (Å) | 0.006 | 0.006 | 0.005 | 0.008 |
| Bond angles (°) | 0.843 | 0.805 | 0.722 | 0.871 |
| Ramachandran plot |  |  |  |  |
| Favored (%) | 99.21 | 100 | 100 | 98.90 |
| Allowed (%) | 0.79 | 0 | 0 | 1.10 |
| Disallowed | 0 | 0 | 0 | 0 |

^a^ Values for the highest-resolution shell are shown in parentheses.

Table S2. The B-values of each amino acid in the TNIP1_pS_122_, TNIP1_pS_123_, and TNIP1_pS_122_pS_123_ peptides.

| peptide | S119 | G120 | T121 | S/SEP122 | S/SEP123 | E124 | F125 | E126 | V127 | V128 |
| --- | --- | --- | --- | --- | --- | --- | --- | --- | --- | --- |
| TNIP1pS_122_ | 65.77 | 57.43 | 51.33 | 41.62 | 44.50 | 37.87 | 25.70 | 25.81 | 25.83 | 29.12 |
| TNIP1pS_123_ |  |  |  | 65.76 | 70.00 | 48.10 | 40.45 | 41.68 | 30.65 | 37.92 |
| TNIP1 pS_122_  pS_123_ |  |  | 61.72 | 40.46 | 58.15 | 43.01 | 30.77 | 28.82 | 31.94 | 35.36 |

Table S3. The thermodynamic parameters of the ITC experiments

| Peptide | FIP200 claw domain | ΔH  kcal/mol | -TΔS  kcal/mol | *K_D_*  μM | N |
| --- | --- | --- | --- | --- | --- |
| TNIP1 | WT |  |  | N. D. |  |
| TNIP1_pS_122_ | WT | -0.66 ± 0.17 | -4.15 | 260 ± 82.3 | 0.96 |
| TNIP1_pS_122_pS_123_ | WT | -0.79 ± 0.07 | -4.19 | 195 ± 30.8 | 1.33 |
| TNIP1_pS_123_ | WT | -2.21 ± 0.13 | -2.89 | 160±15.8 | 1.01 |
|  | R1514A | -1.72 ± 0.19 | -3.31 | 178 ± 40.6 | 1.48 |
|  | D1516A | -1.96 ± 0.09 | -3.08 | 175 ± 14.3 | 1.18 |
|  | Y1564A |  |  | N. D. |  |
|  | Q1566A | -1.13 ± 0.15 | -4.11 | 126 ± 37.6 | 1.49 |
|  | K1568A |  |  | Weak binding |  |
|  | K1568E |  |  | N. D. |  |
|  | K1569A |  |  | N. D. |  |
|  | K1569E |  |  | N. D. |  |
|  | R1573E |  |  | N. D. |  |
|  | F1574A |  |  | N. D. |  |
|  | F1582A |  |  | N. D. |  |
|  | R1584A |  |  | N. D. |  |
| TNIP1^long^ | WT |  |  | Weak binding |  |
| TNIP1_pS_123_ ^long^ | WT | -2.57 ± 0.02 | -3.25 | 45.4 ± 0.99 | 1.03 |
|  | R1514A | -2.85 ± 0.05 | -3.00 | 43.8 ± 2.39 | 0.97 |
|  | D1516A | -2.87 ± 0.03 | -3.11 | 35 ± 1.09 | 0.98 |
|  | Y1564A |  |  | N. D. |  |
|  | Q1566A | -2.22 ± 0.04 | -3.61 | 45 ± 2.35 | 0.96 |
|  | K1568A | -3.18 ± 0.09 | -2.02 | 133 ± 6.67 | 0.93 |
|  | K1568E |  |  | Weak binding |  |
|  | K1569A |  |  | Weak binding |  |
|  | K1569E |  |  | N. D. |  |
|  | R1573E |  |  | N. D. |  |
|  | F1574A |  |  | N. D. |  |
|  | F1582A |  |  | N. D. |  |
|  | R1584A |  |  | N. D. |  |
| CCPG1 | WT | -1.09 ± 0.06 | -4.37 | 84.3 ± 7.2 | 0.96 |
| pCCPG1 | WT | -1.36 ± 0.01 | -5.39 | 9.3 ± 0.5 | 1.10 |

*K_D_*, dissociation constant; N, binding stoichiometry; ΔH, binding enthalpy; -TΔS, binding entropy; N.D., no detectable binding. Each *K_D_* value is presented as fitted value ± error.

Table S4. Peptides used in this study

| Name | Sequence |
| --- | --- |
| TNIP1_FIR | SSGTSSEFEVV |
| TNIP1_pS_123_ | SSGTS-pS-EFEVV |
| TNIP1 _pS_122_ | SSGT-pS-SEFEVV |
| TNIP1 _pS_122_pS_123_ | SSGT-pS-pS-EFEVV |
| TNIP1_FIR^long^ | SSGTSSEFEVVTPEEQ |
| TNIP1_pS_123_^long^ | SSGTS-pS-EFEVVTPEEQ |
| CCPG1 FIR2 | TASDDSDIVTLEPPK |
| pCCPG1 FIR2 | TASDD-pS-DIVTLEPPK |
| FITC-pCCPG1 FIR2 | FITC-TASDD-pS-DIVTLEPPK |

pS: phosphorylated Ser; FITC: fluorescein isothiocyanate.

**Supplementary experimental procedures**

**Peptide preparations**

Peptides were synthesized by GL Biochem (Shanghai), and stock solutions (100 μM to 20 mM) were prepared in buffer B. The sequences of the peptides used were listed in Table S4.

**Fluorescent polarization (FP) assays**

For competitive FP experiments, FIP200 claw domain at 4 μM and 50 nM FITC-pCCPG1 FIR2 in buffer B were mixed with increasing concentrations of unlabeled TNIP1_FIR^long^ or TNIP1 FIR_pS_123_^long^ peptides, respectively. The samples were measured with the CLARIOsta plate reader with FITC-fluorescence polarization filter set. Experiments were performed in triplicate. The inhibition constant of the TNIP1 peptide (Ki) in the ternary system was well fitted under a competitive one-site-binding mode using the following equation (1)

$$A=\frac{2\sqrt{(d^{2}-3e)}cos(Ɵ/3)-d}{3K_{d}+2\sqrt{(d^{2}-3e)}cos(Ɵ/3)-d}(A_{b}-A_{f})+A_{f}$$

where $d= K_{d}+K_{i}+L+x-P$，$e=(x-P)K_{d}+(L-P)K_{i}+K_{d}K_{i}$，$Ɵ= arccos(\frac{-2d^{3}+9de-27f}{2\sqrt{{(d^{2}-3e)}^{3}}})$, $f=-K_{d}K_{i}P$. $A=(I^{\parallel}-I^{\perp})/(I^{\parallel}+2I^{\perp})$; $x$ is the concentration of the TNIP1 FIR peptides; $L$ is the concentration of FITC-pCCPG1 FIR2 peptide; $P$ is the concentration of FIP200 claw domain; $I^{\parallel}$, fluorescence intensity parallel to the plane of exciting light; $I^{\perp}$,fluorescence intensity perpendicular to the plane of exciting light; $A$, anisotropy；$A_{b}$ and $A_{f}$ denote anisotropies of bound and free species, respectively.

**Reference**

1. Roehrl, M. H., Wang Jy Fau - Wagner, G., and Wagner, G. A general framework for development and data analysis of competitive high-throughput screens for small-molecule inhibitors of protein-protein interactions by fluorescence polarization. *Biochemistry* **43**, 16056–16066
